# peIF4E as an independent prognostic factor and a potential therapeutic target in diffuse infiltrating astrocytomas

**DOI:** 10.1002/cam4.817

**Published:** 2016-07-20

**Authors:** Elena Martínez‐Sáez, Vicente Peg, Arantxa Ortega‐Aznar, Francisco Martínez‐Ricarte, Jessica Camacho, Javier Hernández‐Losa, Joan Carles Ferreres Piñas, Santiago Ramón y Cajal

**Affiliations:** ^1^ Department of PathologyVall d'Hebron University HospitalBarcelonaSpain; ^2^ Department of Morphological SciencesUniversitat Autònoma de BarcelonaBarcelonaSpain; ^3^ Department of NeurosurgeryVall d'Hebron University HospitalBarcelonaSpain; ^4^ Department of PathologyCorporació Sanitaria Parc TaulíSabadellSpain

**Keywords:** Astrocytomas, cell signaling, glioblastoma, peIF4E, prognosis

## Abstract

Malignant transformation in tumors is a complex process requiring accumulation of numerous oncogenic abnormalities. Brain tumors show considerable phenotypic and genetic heterogeneity. In a series comprising diffuse infiltrating astrocytomas (DIA) and reactive gliosis, we investigated the main factors associated with signaling pathways. We assessed expression levels and their association with tumor progression and survival. We studied 19 grade II astrocytomas, 25 anaplastic astrocytomas (grade III), 60 glioblastomas (grade IV), and 15 cases of reactive gliosis. Epidermal growth factor receptor (EGFR), pMAPK, 4E‐BP1, p4E‐BP1, pS6, eIF4E, and peIF4E expression levels were evaluated using immunohistochemistry. Expression levels were semiquantitatively evaluated using a histoscore. Immunohistochemistry and PCR were used for IDH1 mutations. Statistical analysis was based on the following tests: chi‐square, Student's *t*, Pearson correlation, Spearman's rho, and Mann–Whitney; ROC and Kaplan–Meier curves were constructed. A significant increase was observed between grades for expression of total and phosphorylated 4E‐BP1 and for eIF4E, Ki67, EGFR, and cyclin D1. Although expression of EGFR, eIF4E, and Ki67 correlated with survival, only peIF4E was an independent predictor of survival in the multivariate analysis. Combining the evaluation of different proteins enables us to generate helpful diagnostic nomograms. In conclusion, cell signaling pathways are activated in DIAs; peIF4E is an independent prognostic factor and a promising therapeutic target. Joint analysis of the expression of 4E‐BP1 and peIF4E could be helpful in the diagnosis of glioblastoma multiforme in small biopsy samples.

## Introduction

Diffuse infiltrating astrocytomas (DIA) are the most common primary brain tumors. The 2007 World Health Organization Classification 1 recognizes three grades of malignancy in these tumors, namely, diffuse astrocytoma (grade II), anaplastic astrocytoma (grade III), and glioblastoma multiforme (GBM, grade IV). Primary GBM appears de novo, with no previous history of brain tumor. Secondary GBM arises from a lower grade DIA and accounts for 5% of all GBMs. In addition, it is found in younger patients, in whom survival is longer. In molecular terms, amplification of epidermal growth factor receptor (EGFR) is the most distinctive marker of primary GBM, and mutations in *IDH*,* TP53*, and *ATRX* are the most distinctive markers of secondary GBM 2. Despite advances in our knowledge of molecular abnormalities in these types of tumor, targets for efficacious treatment have not yet been identified in DIA.

The main cell signaling pathways—Ras‐Raf‐MAPK and PI3K‐AKT‐mTOR—are activated in many types of malignant tumor 3, 4, 5, 6, 7, 8. The Ras‐Raf‐MAPK pathway phosphorylates eukaryotic translation initiation factor 4E (eIF4E) through the kinases MNK1 and MNK2. Activation of the PI3K‐AKT‐mTOR pathway phosphorylates the protein 4E‐BP1 at its six phosphorylation sites. Other kinases such as CDK1 have also been associated with these pathways. Nonphosphorylated 4E‐BP1 binds to eIF4E, thus blocking initiation of protein translation. When 4E‐BP1 is phosphorylated, eIF4E is released and—together with eIF4A and eIF4G—can form the eIF4F complex, which binds the mRNA strand and triggers translation. eIF4E is a key component in the initiation and regulation of translation to eukaryotic cells 4: via its interaction with the 5′ cap structure of messenger RNA, eIF4E binds the mRNA strand to the ribosome. This is the least abundant initiation factor in terms of number of molecules per cell, thus giving it a key role in the regulation of translation. It has a single phosphorylation site, via which it interacts with eIF4G and 4E‐BP1. eIF4G is a major anchor in the recruitment of the ribosome to mRNA, which in addition to eIF4E interacts with other components of translational machinery, such as eIF4A.

Both 4E‐BP1 and eIF4E play a role in the progression and prognosis of various types of tumor 3, 5, 6, 7, 8, 9. Several clinical trials have been performed, and new drugs targeting these convergent factors are currently under study. The objective of the present study was to investigate the main proteins in both signaling pathways in order to identify possible prognostic and treatment factors in DIAs.

## Patients and Methods

### Patient selection

We studied 104 DIAs from 100 patients. The cases were selected retrospectively from the records of the Histopathology Department of Hospital Universitario Vall d'Hebron between 2000 and 2007 and classified histologically following the 2007 World Health Organization Classification of brain tumors. The 104 DIAs comprised 19 (18%) grade II diffuse astrocytomas, 25 (24%) anaplastic astrocytomas, and 60 (57%) glioblastomas. The diagnosis was based on optical microscopy study of hematoxylin–eosin‐stained slices and immunohistochemistry of GFAP, p53, and Ki67. A representative block per case was selected for the study of signaling pathway markers. Most of the selected GBM cases were large specimens because one of our main goals was to study expression throughout the tumor.

We used 15 cases of reactive astrocytosis to evaluate non‐neoplastic activation of the cell proliferation pathways, mainly brain tissue around vascular lesions (nine cases of gliosis surrounding an arteriovenous malformation) and metastatic brain lesions (two cases of metastasis of breast carcinoma and four of lung adenocarcinoma).

Clinical and radiological data (age, sex, tumor site, type of surgical resection) were collected retrospectively from the clinical charts. Survival data were collected using the National Death Index between January and February 2012. Minimum follow‐up was therefore 48 months.

### Reagents and immunohistochemical analysis

A single representative block was selected for each case. Some included brain cortex. The tissue had previously been fixed in 10% buffered formalin and embedded in paraffin blocks. Three‐micrometer thick slices were taken. The antibodies used and their dilutions are shown in Table 1.

**Table 1:** Information on the primary antibodies used in this study

Antibody	Phosphorylation site	Supplier	Host	Dilution
EGFR	Clone 2‐18C9	Dako	Mouse	Prediluted
PTEN	Clone 6 h 2.1	Cascade ABM 2052	Mouse	1:300
pMAPK		Cell Signaling Technology	Rabbit	1:200
4E‐BP1	Ser112	Cell Signaling Technology	Rabbit	1:50
p4E‐BP1	Thr70	Cell Signaling Technology	Rabbit	1:50
pS6		Cell Signaling Technology	Rabbit	1:100
eIF4E	Ser209	Cell Signaling Technology	Rabbit	1:75
peIF4E	Clone EP2151Y	Abcam	Rabbit	1:200
Cyclin D1	Clone SP4	Ventana	Rabbit	Prediluted
Ki67	MIB1	Dako	Mouse	1:100
IDH1 R132H	Clone H09	Master Diagnostica		Prediluted

### Procedures

Immunohistochemistry was performed using the Benchmark XT platform with the ultraView Universal DAB Detection kit (Ventana Medical Systems, Tucson, Arizona) according to previously published protocols 6, 7, 11. Staining of EGFR was performed using the Autostainer Plus (Dako, Tucson, Arizona).

### Immunohistochemistry evaluation

A histoscore (Hscore) was used to perform a semiquantitative evaluation of the antibodies EGFR, pMAPK, p4E‐BP1, 4E‐BP1, pS6, peIF4E, and eIF4E, as previously described 5, 7, 10. The Hscore was calculated by multiplying the intensity score by the percentage of stained cells (from 0% to 100%). The intensity score was ranged from 0 to 3, as follows: 0, no staining, 1, mildly intense staining; 2, moderate intense staining; and 3, strongly intense staining. Levels of Ki67, p53, and cyclin D1 were evaluated as the percentage of cells stained (nuclear staining). The presence or absence of the R132H mutation in *IDH1* was evaluated using immunohistochemistry or PCR 11. Neuronal staining and endothelial staining were used as positive internal controls.

### Statistical analysis

The statistical analysis was performed using IBM SPSS Statistics for Windows, Version 20.0 (IBM Corp, Armonk, New York). Statistical significance was set at *P *<* *0.05. The quantitative variables were age, histologic grade, proliferative activity (Ki67), cyclin D1, p53, and the Hscore of the proteins studied. The qualitative variables were sex, location, laterality, and the presence of the *IDH1* mutation.

Qualitative variables were compared using the chi‐square test. Normally distributed quantitative variables were compared using the *t* test and Pearson correlation; non‐normally distributed quantitative variables were compared using Spearman's rho and the Mann–Whitney test.

Receiver operating characteristic (ROC) curves were used to calculate the cut‐offs that were most sensitive and specific for the diagnosis of high‐grade versus low‐grade disease or GBM versus other tumors in each of the markers. Once the cut‐off was identified, the association with survival was explored using Kaplan–Meier curves. The log‐rank test was used to compare overall differences between the curves. Logistic regression models were constructed to identify which parameters were independently associated with high‐grade disease or GBM.

The multivariate analysis was performed using Cox regression with the aim of finding the independent prognostic value for each of the variables analyzed. The diagnostic impact of the proteins was studied by creating a variable of high‐grade DIA versus low‐grade DIA and a variable of GBM versus non‐GBM.

## Results

### Demographic and clinicopathological characteristics

Clinicopathological data are summarized in Table 2. The differences in age by group were statistically significant (*P *<* *0.001). Mortality was 63.1% for grade II lesions, 56% for grade III lesions, and 83.7% for GBM. Mean survival time was 1007 days (1567 for grade II, 1302 for grade III, and 520 for GBM). Significant differences in survival were found according to histological grade (*P *<* *0.001) and age (*P *<* *0001).

**Table 2:** Clinicopathological data

Characteristics	Total neoplastic cases (%)	Grade II	Grade III	Grade IV	Gliosis
Total cases	104 (100)	19 (19)	25 (24)	60 (57)	15
Median age (years)	53 ± 15.7	43.53 ± 17.9	43.1 ± 14.3	60.3 ± 11.2	46.3
Gender					
Male	37 (37.4)	12 (60)	21 (84%)	33 (55%)	10
Female	61 (62.6)	7 (40)	4 (16%)	27 (45%)	5
Location					
Frontal lobe	35 (33.7)	8	9	18	NA
Temporal lobe	30 (28.8)	6	4	20	NA
Parietal lobe	8 (7.7)	2	2	4	NA
Occipital lobe	3 (2.9)	0	0	3	NA
Other location	13 (12.5)	2	5	6	NA
Location NA	15 (14.4)	1	5	9	NA
Surgical procedure					
Lobectomy	34 (32.7)	7	10	17	NA
Subtotal resection	37 (35.6)	6	2	29	NA
Open biopsy	23 (22.1)	2	7	14	NA
Stereotactic biopsy	10 (9.6)	4	6	0	NA
OS (days)	1007	1567.8	1302.2	520.5	NA

NA, not applicable/not available; OS, overall survival.

### p4E‐BP1

**Figure 1:**
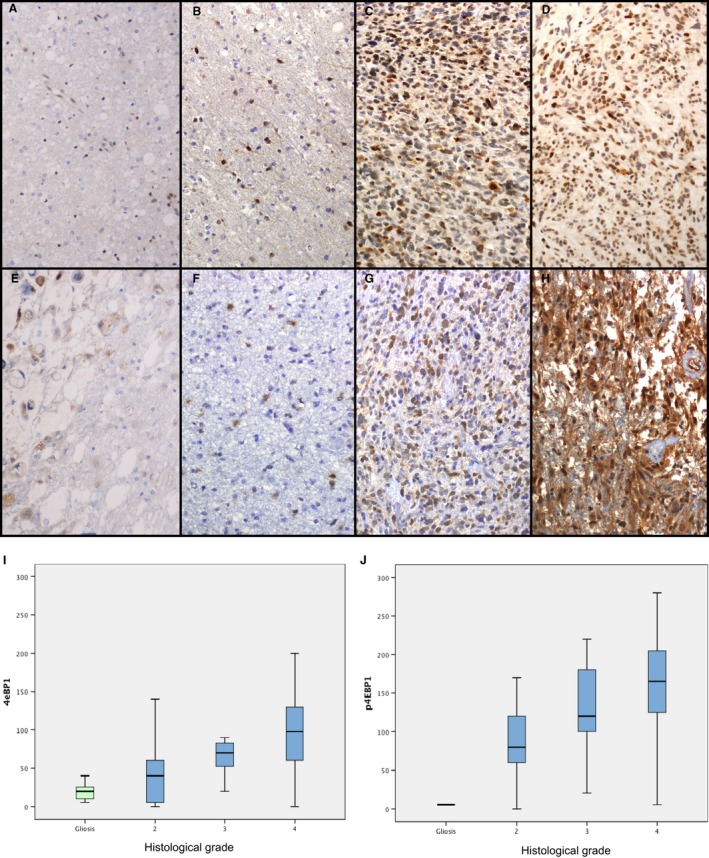
Expression of 4E‐BP1 (A–D) and p4E‐BP1 (E–H) in gliosis (A, E), diffuse astrocytoma (B, F), anaplastic astrocytoma (C, G), and glioblastoma multiforme (GBM) (D, H). Significant differences were found in 4E‐BP1 (I) and 4E‐BP1 (J) expression between grades, *P *<* *0.001.

p4E‐BP1 was expressed in the cytoplasm of tumor cells. A significant correlation was found between expression of p4E‐BP1 and tumor grade (*P *<* *0.001, Fig. 1F, G, H, and J). As for the impact of evaluating expression of p4E‐BP1 on diagnosis, we found cut‐off points revealing significant differences between groups, with high sensitivity (72.9%) that enabled us to differentiate GBM from astrocytomas of other grades. No significant differences in survival were found between groups with higher or lower p4E‐BP1 levels.

### 4E‐BP1

Expression of total 4E‐BP1 in the cytoplasm of neoplastic cells increased with tumor grade (*P *<* *0.001, Fig. 1B, C, D, and I). As for the impact of evaluating expression of 4E‐BP1 on diagnosis, we found cut‐off points that indicated significant differences between groups, with better sensitivity for differentiation between high‐grade and low‐grade astrocytomas (70.5%) than in the diagnosis of GBM (60.3%) and a high positive predictive value (PPV, 94.8%). No significant differences in survival were found between groups with higher or lower 4E‐BP1 levels.

### peIF4E

**Figure 2:**
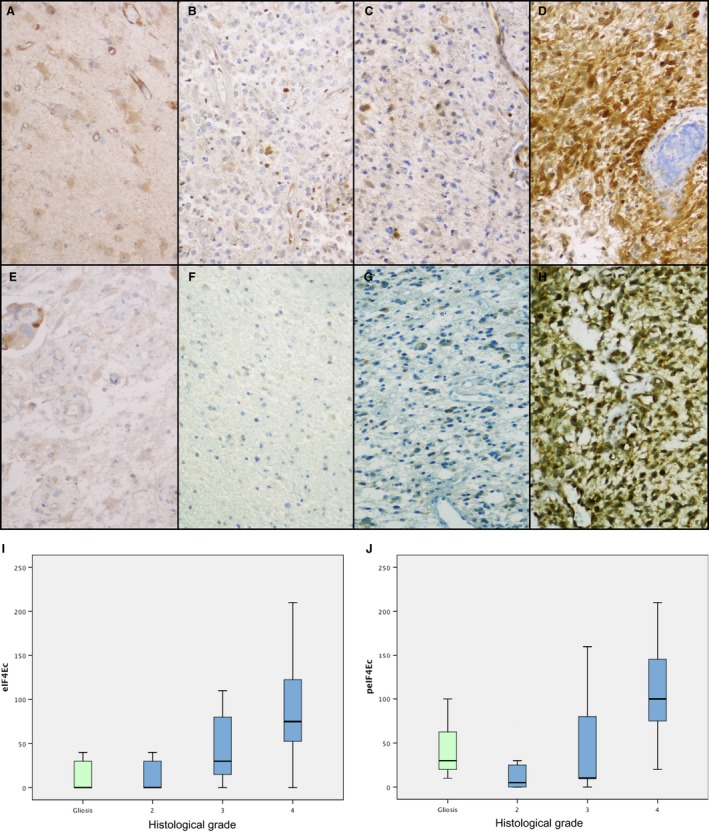
Expression of peIF4E (A–D) and eIF4E (E–H) in gliosis (A, E), diffuse astrocytoma (B, F), anaplastic astrocytoma (C, G), and glioblastoma multiforme (GBM) (D, H). Significant differences (*P *<* *0.001) were found in the expression of eIF4E (I) and peIF4E (J).

**Figure 3:**
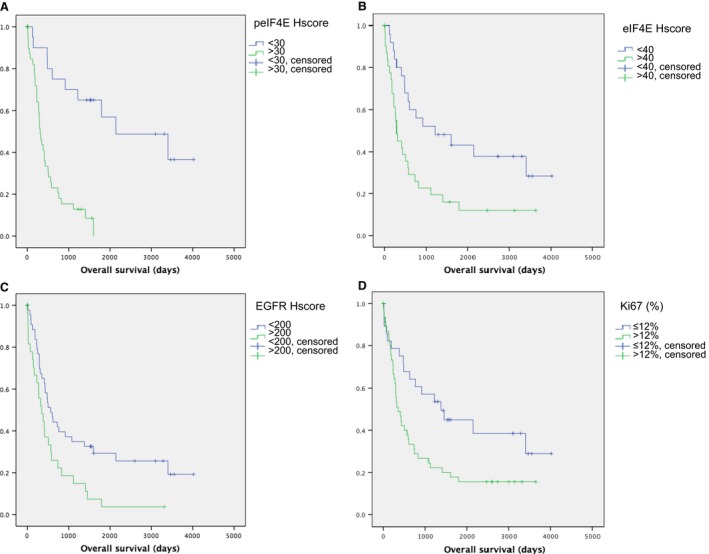
Significant differences in survival based on peIF4E score (A, *P *<* *0.001), eIF4E score (B, *P *=* *0.006), epidermal growth factor receptor (EGFR) score (C, *P *=* *0.005), and Ki67 (D, *P *=* *0.014).

Phosphorylated eIF4E was expressed in the cytoplasm of tumor cells. Significant differences were found in expression of peIF4E for the different tumor grades (*P *<* *0.001, Fig. 2F, G, H, and J). With respect to diagnosis, the cut‐off points indicated significant differences between the groups, with better sensitivity in the diagnosis of GBM (94.3%) than in differentiation between high‐grade and low‐grade astrocytomas (81.4%), and a high PPV in both cases (96.6% in high grade vs. low grade and 87.7% in GBM vs. other grades). Phosphorylated eIF4E also had a significant impact on survival (Fig. 3A), even when the groups were stratified by grade. Multivariate analysis using Cox regression showed that peIF4E was the only independent predictor of survival.

### eIF4E

Cytoplasmic expression of eIF4E showed a statistically significant association with tumor grade (*P *<* *0.001, Fig. 2B, C, D, and I). As for the diagnostic value of the eIF4E Hscore, we found the same cut‐off with better sensitivity and specificity for the diagnosis of high‐grade DIA than for the diagnosis of low‐grade DIA, and for the diagnosis of GBM than for the diagnosis of DIA of other grades. In the first case, the PPV of eIF4E levels reached 100%.

Expression of eIF4E can also predict prognosis, with significant differences in survival between patients who exceed the cut‐off (mean survival: 814 days) compared with those who do not (mean survival: 1911 days; *P *=* *0.006, Fig. 3B). This finding holds for the whole population, but the differences are lost when patients are stratified by grade.

### EGFR

EGFR was expressed in cell membranes, and the Hscore increased significantly with grade (*P *=* *0.015). We found the same cut‐off for separating low‐grade and high‐grade DIA (PPV, 93.8%) and for diagnosing GBM compared with other types of DIA (PPV, 81.3%). EGFR levels also had an impact on survival: patients with EGFR levels higher than 200 lived for 616 days, whereas those with lower levels lived for 1579 days (*P *=* *0.005, Fig. 3C).

### pS6

pS6 was expressed mainly in the cytoplasm. With respect to the role of pS6 in the diagnosis of DIA, after finding the cut‐off with the greatest sensitivity for separating high‐grade and low‐grade DIA and for separating GBM from the remaining types of DIA, we observed that the differences between the groups were not real (*P *=* *0.163 and 0.076). Similarly, pS6 had no impact on survival in our series.

### pMAPK

**Figure 4:**
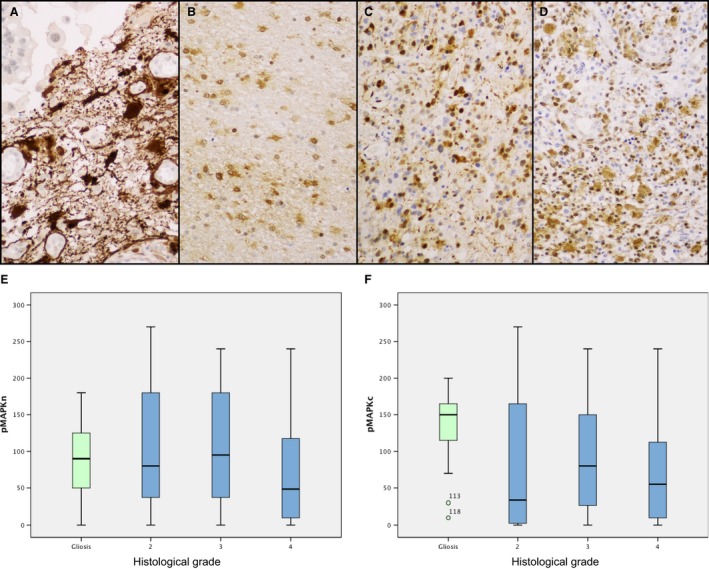
Expression of pMAPK in gliosis (A), diffuse astrocytoma (B), anaplastic astrocytoma (C), and glioblastoma multiforme (GBM) (D). No significant differences between grades were found in nuclear (E) and cytoplasmic (F) pMAPK expression.

Staining was positive in the cytoplasm and nucleus of tumor cells. No correlation was found between pMAPK expression and histological grade (Fig. 4A–F). No impact on diagnosis or on survival was found for pMAPK levels.

### Cyclin D1

Cyclin D1 was expressed in the nucleus of tumor cells. The degree of expression was greater in the DIA group (*P *=* *0.004), the GBM group (*P *<* *0.001), and the anaplastic astrocytoma group (*P *=* *0.039) than in the gliosis group. Significant differences were found between expression of cyclin D1 in diffuse astrocytoma and in high‐grade DIA (*P *=* *0.001).

As for the impact of cyclin D1 on diagnosis, the cut‐off found was the same (statistically significant), with better sensitivity and specificity in the diagnosis of high‐grade DIA compared with low‐grade DIA and in the diagnosis of GBM compared with other DIAs. However, no statistically significant differences in survival were found between the groups based on these cut‐offs.

### Ki67

The tumor proliferation index increased with tumor grade (*P *<* *0.001). The cut‐off we found for Ki67 enabled us to differentiate between high‐grade and low‐grade DIA and between GBM and other grades, with good PPV and sensitivity. In addition, significant differences in survival were found between patients with Ki67 above the cut‐off (mean: 942 days) and patients with a lower proliferative index (mean: 1936 days, *P *=* *0.014, Fig 3D).

### Summary and combined evaluation of various proteins (nomograms)

**Figure 5:**
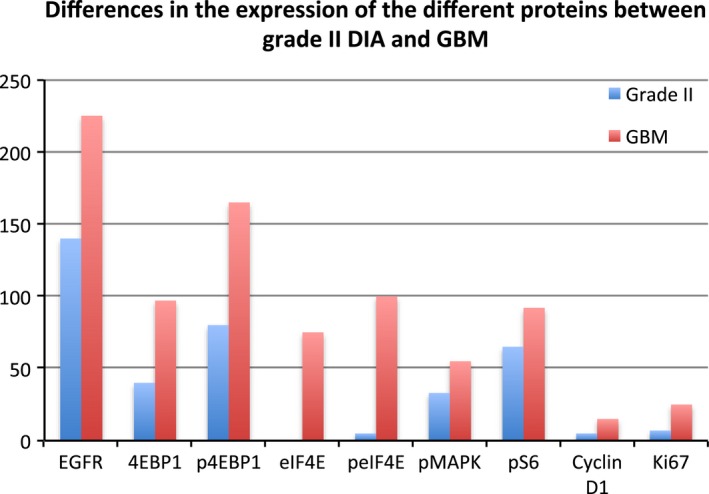
Differences in the expression of the different studied proteins in grade II astrocytomas and GBM (mean levels).

Hscore values are summarized in Table 3 (median and confidence interval). As shown in Figure 5, central factors that control protein translation are overexpressed in high‐grade gliomas. We applied a logistic regression model to analyze which variables were independently associated with a diagnosis of high‐grade DIA or a diagnosis of GBM. The cut‐off points that gave the best combinations were as follows: (1) 4E‐BP1 >85 and peIF4E >30: diagnosis of GBM with a PPV of 100% (*P *<* *0.001). (2) Cyclin D1 > 2% and peIF4E >30: diagnosis of high‐grade DIA with a PPV of 98.7% (*P *<* *0.001). (3) peIF4E <30 and Ki67 < 12%: exclusion of GBM, with a negative predictive value of 100% (*P *<* *0.001).

**Table 3:** Hscore (median values and confidence interval) for p4E‐BP1, 4E‐BP1, peIF4E, eIF4E, EGFR, pS6, and pMAPK in gliosis cases, grade II diffuse astrocytomas, AA, and GBM. Cyclin D1 and Ki67 are expressed as percentages

	Gliosis	DA	AA	GBM	Median	CI [5–95%]	Median	CI [5–95%]
	Median	CI [5–95%]	Median	CI [5–95%]				
p4E‐BP1	5	0–20	80	0–185	120	20–220	165	30–275
4E‐BP1	20	5–50	40	5–130	70	5–170	100	10–200
peIF4E	30	10–90	5	0–110	10	0–130	100	30–190
eIF4E	5	5–60	5	5–40	30	5–160	75	5–185
EGFR	‐a	‐a	140	0–225	140	0–300	225	15–300
pS6	120	30–170	65	5–230	40	5–230	90	20–240
pMAPK^b^	150	0–188	35	0–185	80	0–215	55	0–200
Cyclin D1	5	0–30	5	0–20	15	0–50	15	0–40
Ki67	1	0–3	5	1–15	15	2–45	25	5–75

DA, diffuse astrocytoma; AA, anaplastic astrocytoma; GMB, glioblastoma multiforme; CI, confidence interval. EGFR, epidermal growth factor receptor.

a The EGFR study was not performed in cases of gliosis.

b pMAPK values correspond to cytoplasmic pMAPK.

### Secondary glioblastomas

No significant differences were found in levels of protein expression in the signaling pathways in the two cases of confirmed secondary GBM with respect to the remaining cases of GBM in the series (see Data S1).

### Expression of cell signaling factors in gliosis

As a rule, the expression of most of the proteins studied was mild. Levels of p4E‐BP1 (Fig. 1E), 4E‐BP1 (Fig. 1A), peIF4E (Fig. 2E), eIF4E (Fig. 2A), and cyclin D1 and the Ki67 proliferative index were significantly lower in gliosis than in neoplasm in the whole group (*P *=* *0.004 for cyclin D1, *P *<* *0.001 for the other proteins). These differences held for all three grades in p4E‐BP1 and Ki67. As for 4E‐BP1, eIF4E, and cyclin D1, expression in gliosis was significantly lower than in high‐grade DIA but not grade II astrocytoma. Expression of peIF4E in gliosis was similar to that found in anaplastic astrocytoma and higher than that observed in grade II astrocytomas.

Cytoplasmic expression of pMAPK in gliosis was significantly more pronounced than in GBM (*P *=* *0.003), although no differences were found between gliosis and grade II and grade III astrocytoma. No differences were found regarding nuclear expression of pMAPK. pS6 levels in gliosis were similar to those detected in tumors, with no significant differences between the tumor group and the nontumor group.

## Discussion

Cell signaling coordinates basic cellular activities and is based on growth factor membrane receptors and intracellular signaling pathways such as RAS‐Raf‐MAPK, STAT3, and PI3K‐AKT‐mTOR. We showed that the levels of these factors (p4E‐BP1, peIF4E) increase during tumor progression and that peIF4E, age, and histologic grade were independent prognostic factors in the multivariate analysis. These results are consistent with those of previous studies in several types of carcinomas, where p4E‐BP1, eIF4E, and peIF4E were overexpressed, associated with a poorer prognosis, and considered critical “funnel factors” in cell signaling 3, 5, 6, 7, 8, 12, 13, 14.

The protein 4E‐BP1 and its phosphorylated form have received little attention in the context of DIAs 15, 16, 17. In the present study, we found a correlation between tumor grade and levels of expression of the total and phosphorylated forms of 4E‐BP1. Similar results were previously reported in a study on p4E‐BP1 by Korkolopoulou et al. 15, who examined a series of 111 DIAs and found a correlation between expression of p4E‐BP1 and tumor grade. Of the many factors involved in the mTOR pathway analyzed by this group, p4E‐BP1 was the only one that showed a correlation with MAPK. The authors reported that p4E‐BP1 predicted a worse prognosis, regardless of the mutational status of *IDH1*. Even though we found that p4E‐BP1 levels correlated with survival, p4E‐BP1 was not an independent prognostic factor in the present series. In addition, no association was found with the mutational status of *IDH1*, although it is important to remember that the number of GBMs with the R132H mutation in our series is insufficient to draw statistical comparisons. Ermoian et al.^16^ studied p4E‐BP1 levels using immunoblotting in 71 DIA specimens distributed homogeneously into three groups according to tumor grade (28 grade II, 17 anaplastic astrocytoma, and 26 GBM), with a control group of 16 non‐neoplastic specimens. The results reported were similar to ours when the four groups were compared (statistically significant correlation between levels of p4E‐BP1 and progression of tumor grade); however, statistical significance disappeared when the control group was removed. Differences were found in the expression of pAKT between the group formed by control cases and low‐grade tumors and the group comprising high‐grade tumors. As for the mTOR complex, the authors found reduced levels of the mTOR‐suppressing proteins hamartin and tuberin (TSC1 and TSC2) as the tumor grade increased. This halt in the inhibition of mTOR enables it to act on 4E‐BP1 by phosphorylating it and thus increasing p4E‐BP1 levels. An association was found between the increase in pAKT and p4E‐BP1, but not between pAKT and S6. Similarly, no impact on survival was recorded. In their study of 29 cases of GBM, Riemenschneider et al. 17 reported that activation of AKT did not imply a statistically significant increase in p4E‐BP1 levels. These findings support the possibility that kinases other than mTOR (like CDK1) could phosphorylate 4E‐BP1 6, 18.

Overexpression of eIF4E has been reported in cancer of the lung, breast, prostate, bladder, cervix, ovary, thyroid, and head and neck, as well as in hematologic neoplasms 19. It is associated with tumor progression and poor prognosis in cancer of the breast 20, bladder 21, 22, prostate 23, 24, and cervix 25, and in lung adenocarcinoma 26, 27. Consistent with reports on these tumors, we observed overexpression of eIF4E in astrocytoma with respect to gliosis. Only one study has compared overexpression of eIF4E in high‐grade astrocytoma (10 cases of anaplastic astrocytoma and GBM) and non‐neoplastic brain parenchyma 28. The statistically significant correlation between more marked expression of eIF4E with higher tumor grade and poor prognosis in DIA had not been previously reported. In other tumors, expression of eIF4E was reported to be more marked in neoplastic lesions than in preneoplastic lesions (e.g., higher expression of eIF4E in adenocarcinoma than in adenomatous polyps in the colon 26 or in infiltrating carcinoma compared with benign tumors of the head and neck 29).

The biological significance of phosphorylation of eIF4E and its effect on translation is not well understood. Some studies indicate that phosphorylation of eIF4E reduces the affinity of this protein for the 5′ end of the mRNA strand 30. This phosphorylation does not seem to play an important role in the normal functioning of the healthy cell, although it does appear to play a key role in tumors and has been associated with increased synthesis of several proteins involved in the development of neoplasms (McI‐1, MMP‐3, and cyclin D1) 24. Similarly, peIF4E has been shown to confer resistance in situations of cellular stress, such as starving and genomic damage 31.

Phosphorylation of eIF4E increases in the initial stages of the development of tumors of the breast, colon, stomach, and lung. It increases in prostate cancer and is associated with neoplastic growth not related to androgens 32, although differences in peIF4E expression have not been reported between tumors with and without nodal metastasis 33. Fan et al. report overexpression of peIF4E using immunohistochemistry in a case of astrocytoma from a small series of five cases with no expression of the protein in healthy brain tissue. In the present study, we observed that the phosphorylated form of eIF4E increased with tumor grade. Marked expression of this protein was the only independent prognostic factor among those involved in the cell signaling pathways analyzed.

### Diagnostic and therapeutic implications

Biopsy specimens for the diagnosis of DIA are often small, and the histologic characteristics of the material sent for analysis do not always coincide with the radiologic appearance of the lesion. In such cases, molecular data such as amplification of EGFR can be useful in clinical decision making. We asked whether the proteins investigated in the present study had a predictive value in the diagnosis of high‐grade astrocytoma and GBM and an impact on survival. Thus, even if histologic criteria are not fulfilled, a DIA lesion showing expression of 4E‐BP1 and peIF4E with Hscores >85/300 and 30/300, respectively, could suggest a diagnosis of GBM (with a positive predictive value of 100%, *P *<* *0.001). In such cases, it would be necessary to evaluate the risk–benefit ratio of a new biopsy.

Phosphorylation of 4E‐BP1 and eIF4E are examples of activation of multiple biochemical pathways upstream, where many oncogenic abnormalities may be activated. Phosphorylation of eIF4E was recently shown to confer greater aggressivity in cells and in the development of metastasis in murine models 34. It is also associated with resistance to oxidative stress, nutrient deprivation, and cytotoxic stress 31, 34. Therefore, inhibition of phosphorylation was proposed as a therapeutic target. In clinical terms, attempts have been made to inhibit expression of eIF4E with antisense oligonucleotides, which block binding to eIF4G and inhibit phosphorylation with MAPK inhibitors and with ribavirin 21, 32, 35. MNK inhibitors such as CGP 57380 and cercosporamide block growth of GBM in animal models 36 and yield better results when combined with mTOR inhibitors. In vitro blockade of eIF4E by CGP 57380 is sufficient to reduce cell migration and cellular resistance to neoplastic cells and chemotherapeutic agents 21, 31, 37. Inhibiting phosphorylation of eIF4E by inhibition of MAPK and MNKs could then block the proliferative effect of eIF4E. It could therefore prove useful for sensitizing tumor cells to anticancer agents, thus highlighting the usefulness of combining eIF4E phosphorylation inhibitors with other antitumor agents 31.

## Conflict of Interest

None declared.

## Supporting information


**Data S1.** Secondary GBM.
