# Supplementary material for: peIF4E as an independent prognostic factor and a potential therapeutic target in diffuse infiltrating astrocytomas
Source: Cancer Med. 2016 Jul 20;5(9):2501–12. doi: 10.1002/cam4.817 (PMC5055163; doi:10.1002/cam4.817)
Supplement: Supplementary file 1 — Data S1. Secondary GBM. [file CAM4-5-2501-s001.docx]

SUPPLEMENTARY DATA

Secondary GBM

Immunohistochemistry to investigate the *IDH1* R132H mutation in the 60 cases of GBM revealed 2 mutated cases. One was a man aged 35 years with a previous biopsy of anaplastic astrocytoma (thus confirming secondary GBM) and overexpression of p53 (60%). The patient died 5 years after the diagnosis of GBM. The other patient was a 31-year-old woman with no evidence of a previous low-grade lesion or overexpression of p53 who died immediately after surgery.

Immunohistochemistry revealed >40% overexpression of p53 in 9 patients who did not carry R132H. *IDH* mutational status was assessed using PCR, although no mutations were detected. Seven of the 9 patients were aged over 55 years, with no previous lesions (therefore, not very likely to be secondary GBM). The other 2 cases were patients aged 35 and 38 years (expression of p53 of 90% and 70%, respectively), who survived for 2 and 3 years.
